# TNF-α stimulates endothelial palmitic acid transcytosis and promotes insulin resistance

**DOI:** 10.1038/srep44659

**Published:** 2017-03-17

**Authors:** Wenjing Li, Xiaoyan Yang, Tao Zheng, Shasha Xing, Yaogong Wu, Fang Bian, Guangjie Wu, Ye Li, Juyi Li, Xiangli Bai, Dan Wu, Xiong Jia, Ling Wang, Lin Zhu, Si Jin

**Affiliations:** 1 Department of endocrinology, Institute of geriatric medicine, Liyuan Hospital, Tongji Medical College, Huazhong University of Science and technology, 430077, Wuhan, Hubei, China; 2 Department of Pharmacology, School of basic medicine, Tongji Medical College, Huazhong University of Science and technology, 430030, Wuhan, Hubei, China; 3 Hubei Key Laboratory of Drug Target Research and Pharmacodynamic Evaluation, Tongji Medical College, Huazhong University of Science and technology, 430030, Wuhan, Hubei, China

## Abstract

Persistent elevation of plasma TNF-α is a marker of low grade systemic inflammation. Palmitic acid (PA) is the most abundant type of saturated fatty acid in human body. PA is bound with albumin in plasma and could not pass through endothelial barrier freely. Albumin-bound PA has to be transported across monolayer endothelial cells through intracellular transcytosis, but not intercellular diffusion. In the present study, we discovered that TNF-α might stimulate PA transcytosis across cardiac microvascular endothelial cells, which further impaired the insulin-stimulated glucose uptake by cardiomyocytes and promoted insulin resistance. In this process, TNF-α-stimulated endothelial autophagy and NF-κB signaling crosstalk with each other and orchestrate the whole event, ultimately result in increased expression of fatty acid transporter protein 4 (FATP4) in endothelial cells and mediate the increased PA transcytosis across microvascular endothelial cells. Hopefully the present study discovered a novel missing link between low grade systemic inflammation and insulin resistance.

Metabolic cardiovascular disorders including type 2 diabetes mellitus (T2DM) and atherosclerosis are intimately connected to perturbations in the systemic lipid balance. Excessive lipid deposition in peripheral tissues impairs insulin sensitivity and hampers glucose uptake by tissue cells; therefore it has been proposed to contribute to the development of insulin resistance, the key event in T2DM^1^,^2^. The presence of excessive amounts of lipids in the heart, a metabolic organ, is a strong predictor of heart disease^3^,^4^.

Elevated plasma triglyceride (TG) or free fatty acid (FFA) are recognized risk factors of diabetes, because of their actions on insulin sensitivity in peripheral tissues^5^,^6^,^7^,^8^,^9^,^10^,^11^,^12^,^13^. In plasma, fatty acid (FA) molecules are bound to albumin molecules and become water-soluble. To act on cells, FA molecules must pass through the microvascular endothelial barrier. In the luminal side of endothelial cells, the FFA-albumin complex binds with FA transporter protein 4 (FATP4), is transported into the cellular cytosol and then is exported outside the cell to the basolateral side, a physiological process termed transcytosis that guarantees a supply of FA to tissue cells. However, excessive FA transport results in lipid accumulation in peripheral tissues causing blunted insulin signaling and impaired glucose uptake, which are key components of several chronic syndromes associated with obesity such as T2DM and metabolic syndrome^1^,^14^. Previous research demonstrated that increased lipid levels in the heart result in deficiencies of myocardium contraction and left ventricular dysfunction^15^. Saturated long-chain FA such as palmitic acid (PA) and stearic acid were potent inducers of these dysfunctional effects^15^,^16^.

The endothelium is at the interface of blood and tissue and its functions are closely regulated by changes in the metabolism. T2DM and metabolic syndrome cause endothelial dysfunction^17^. Under physiological conditions, β-oxidation of FA accounts for 70–80% of ATP generation in the heart. Because FAs have low aqueous solubility, they are transported as albumin-bound complexes of lipoproteins in the circulation. Furthermore, they cannot directly pass through the continuous capillary of heart^15^. VEGF-B, PPAR-γ and apelin regulate the FA transport gatekeeper functions of endothelial cells^18^. FA transport through membrane occurs *via* a membrane protein-mediated mechanism, rather than by diffusion or flip-flop mechanisms^19^. FA transport proteins (FATPs, solute carrier family 27), a family of transmembrane proteins, are directly involved in FA transport and over-expression of FATPs enhances FA uptake^19^,^20^. FATP mRNA contains binding sites for SP1, C/EBP, NF-κB and AP-2^21^. In endothelial cells, FATP3 and FATP4 have independent and synergistic activities for the uptake of long chain FA (LCFA)^15^,^18^,^22^. Hagberg *et al*. reported paracrine VEGF-B signaling controls endothelial transcytosis of FA via transcriptional regulation of FATP3 and FATP4 in the heart^18^,^23^.

Recent studies have suggested low-grade systemic inflammation is closely correlated with metabolic syndrome. Low-grade chronic inflammation is characterized by a 2-3-fold increase in the systemic concentrations of cytokines such as TNF-α, IL-6, and C-reactive protein (CRP)^24^. Excessive proinflammatory cytokines may have significant impact on promoting endothelial conversion from a quiescent phenotype toward an activated one. These milieus probably place a significant burden on the endothelial signaling mechanisms that mediate the normal uptake of circulating FAs leading to accumulation in non-adipose tissues, such as the heart and skeletal muscles^18^. In rodent obesity model^25^ and obese humans^26^, the systemic levels of proinflammatory factor- TNF-α are significantly increased^27^. TNF-α perturbs endothelial function and up-regulates LDL transport through endothelia, thereby accelerating the development of atherosclerosis^28^. TNF-α is also an important link between obesity and insulin resistance. A study in TNF-α^−/−^ mice indicated TNF-α deficiency significantly ameliorated insulin resistant in obese mice but not in lean mice^29^.

Fatty heart correlates with left ventricle dysfunction and systemic or local inflammation in several studies^30^. We observed increased FATP4 expression in cardiac microvessels and the activation of NF-κB in HFD-induced obese mice with impaired insulin sensitivity (Supplemental data). These results suggested that enhanced PA transport across cardiac microvascular endothelial cells (CMECs) and increased TNF-α level might be associated with cardiac insulin resistance.

Autophagy is a process of self-cannibalization for the degradation and recycling of long-lived proteins, mature ribosomes and entire organelles. Research in the last decades have indicated that dysregulation of autophagy might contribute to the development of metabolic disorders, including insulin resistance, diabetes mellitus, obesity, and atherosclerosis^31^. However the levels and role of autophagy in the diabetic heart are unclear^32^. A recent study concluded that autophagy is increased in the diabetic heart regardless of the functional status of the myocardium^33^. Meyer *et al*. reported a dual role of autophagy in the cardiovascular system because basal autophagy protected hearts from oxidative stress and ER stress, but excessively stimulated autophagy caused autophagic death^34^. Cao *et al*. indicated that suppressing autophagy is beneficial for cardiac hypertrophy^35^.

Many studies have reported lipotoxicity or proinflammatory factors are associated with insulin resistant. However, whether or not the proinflammatory factor can induce cardiac insulin resistant through the up-regulation of FA transport across the CMECs and thus to accelerate the lipid deposition in cardiomyocytes, is not clear. In the present study, PA and TNF-α were deemed the representative of FA and proinflammatory factors, respectively. We hypothesized that TNF-α could increase the transcytosis of PA across CMECs and therefore facilitate PA-induced cardiac insulin resistance. The specific roles of autophagy and NF-κB in TNF-α induced PA transcytosis were investigated.

## Results

### Establishment of an *in vitro* PA transcytosis model and determination of PA transcytosis across CMECs

**Figure 1:**
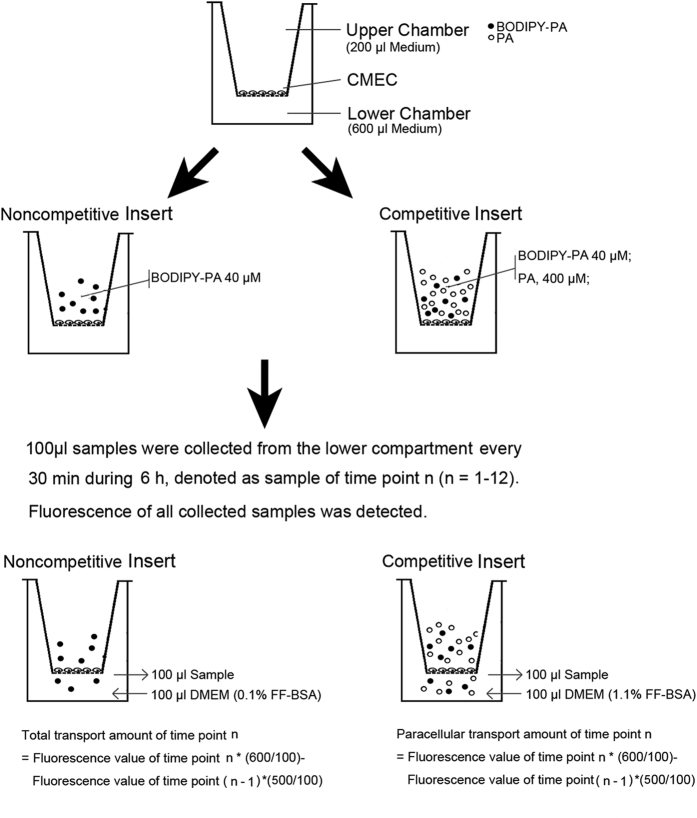
Schematic diagram of the BODIPY-PA transcytosis model. Transport of BODIPY-PA through the CMEC monolayer was performed at 37 °C. Forty μM BODIPY-PA was added into the upper side of all inserts, and tenfold excess of PA was added into the upper side of the competitive inserts for 6 h. Samples was collected from the outer chambers and the fluorescence was measured.

**Figure 2:**
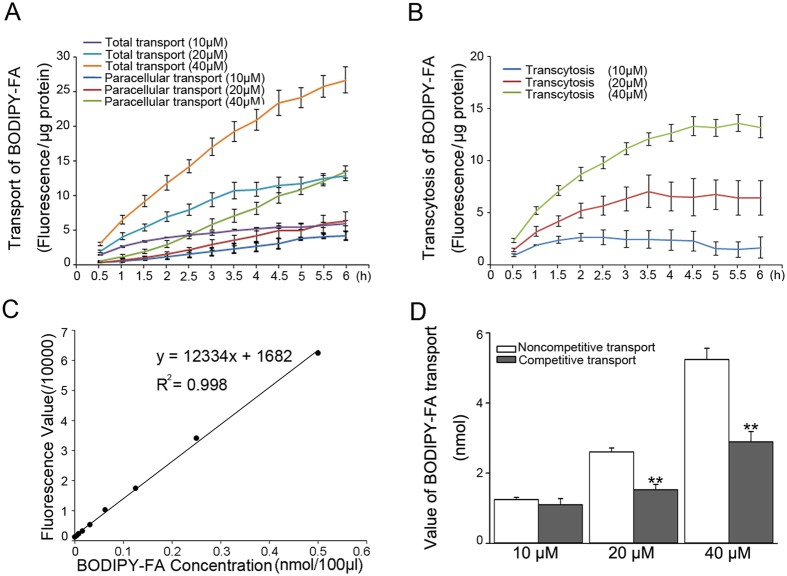
Analysis of BODIPY-PA, an PA analogue, transcytosis in an *in vitro* model. Transcytosis of BODIPY-PA (**B**) was calculated by subtracting paracellular transport from total transport (**A**). Standard curve of fluorescence value related to BODIPY-PA concentration was constructed (**C**). Value of transport during 6 h in non-competitive and competitive groups was calculated by the standard curve (**D**). **P < 0.01 versus non-competitive groups, by 2-tailed Student’s test; *n* = 3.

In this study, we established an *in vitro* model of transcytosis across CMECs monolayers (Fig. 1) to examine the underlying mechanism of PA transcytosis. As shown in Fig. 2A and B, C1-BODIPY-C12 (BODIPY-PA), a fluorescent PA analogue, transport increased in response to the time and concentration of BODIPY-PA used. Paracellular transport was significantly lower than total transport. We constructed a standard curve of BODIPY-PA fluorescence values to confirm this model. As shown in Fig. 2C, there was a linear correlation between the fluorescence value and the concentration of BODIPY-PA. The amount of BODIPY-PA transported through endothelial cell monolayers was calculated by the standard curve shown in Fig. 2D. Amounts in the noncompetitive group (about 2.5 nmol with 20 μM and 5 nmol with 40 μM BODIPY-PA) were significantly greater than in competitive group (about 1.5 nmol with 20 μM and 3 nmol with 40 μM BODIPY-PA). The transcytosis model with 40 μM BODIPY-PA was used in the following experiments.

### TNF-α stimulated PA transcytosis is dependent on autophagy and NF-κB signaling

**Figure 3:**
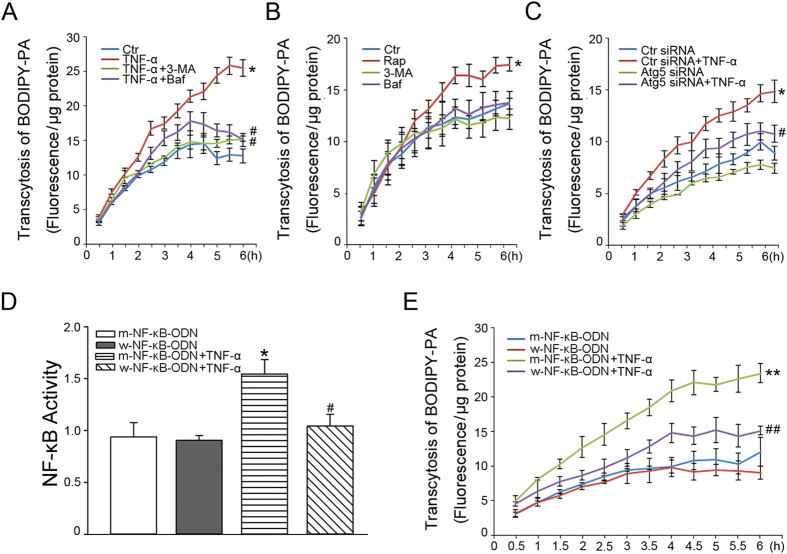
Autophagy and NF-κB are involved in TNF-α–induced PA transcytosis. (**A**) CMECs were pretreated with 5 mM 3-MA or 2 nM Baf for 30 min, and then exposed to 10 ng/ml TNF-α for 18 h. (**B**) CMECs were incubated with 5 mM 3-MA, 2 nM Baf or 10 nM Rap) for 18 h. BODIPY-PA transcytosis across CMECs was measured (*n* = 3); *P < 0.05 versus control (Ctr); ^#^*P* < 0.05 versus TNF-α. (**C**) CMECs were transfected with Ctr siRNA or Atg5 siRNA for 48 h, followed by 10 ng/ml TNF-α incubation for 18 h (*n* = 4); *P < 0.05 versus Ctr siRNA; ^#^*P* < 0.05 versus Ctr siRNA+TNF-α. CMECs were transfected with w-NF-κB-ODN or m-NF-κB-ODN for 24 h. Then NF-κB activity in CMECs exposed to 10 ng/ml TNF-α for 30 min (**D**) and BODIPY-PA transcytosis across CMECs exposed to 10 ng/ml TNF-α for 18 h (**E**) was measured (*n* = 3); *P < 0.05 or **P < 0.01 versus m-NF-κB-ODN; ^#^*P* < 0.05 or ^##^*P* < 0.01 versus m-NF-κB-ODN+TNF-α. NF-κB activity was evaluated by one-way ANOVA and BODIPY-PA transcytosis was evaluated by MNOVA.

We then tested the effects of adding TNF-α to the model of BODIPY-PA transcytosis. Exposure to 10 ng/ml TNF-α for 18 h increased BODIPY-PA transcytosis (Fig. 3A). To clarify further the role of autophagy in TNF-α–induced transcytosis, we pretreated CMECs with autophagy inhibitor 3-MA or Bafilomycin (Baf) for 30 min, followed by 10 ng/ml TNF-α for 18 h. Pretreatment with 3-MA or Baf attenuated TNF-α–induced transcytosis, whereas 3-MA or Baf alone had no effect on BODIPY-PA transcytosis (Fig. 3A,B). Simalily, Autophagy related gene 5 (Atg5) siRNA also attenuated TNF-α–induced transcytosis (Fig. 3C). In addition, incubation with mTOR inhibitor rapamycin (Rap) to stimulate autophagy also enhanced BODIPY-PA transcytosis (Fig. 3B). As shown in Fig. 3D, TNF-α upregulated NF-κB activity. To confirm the role of NF-κB in TNF-α–induced transcytosis, NF-κB activity was suppressed with w-NF-κB-ODN. w-NF-κB-ODN inhibited the up-regulation of NF-κB activity and PA transcytosis induced by TNF-α, but m-NF-κB-ODN had no effect (Fig. 3D,E).

**Figure 4:**
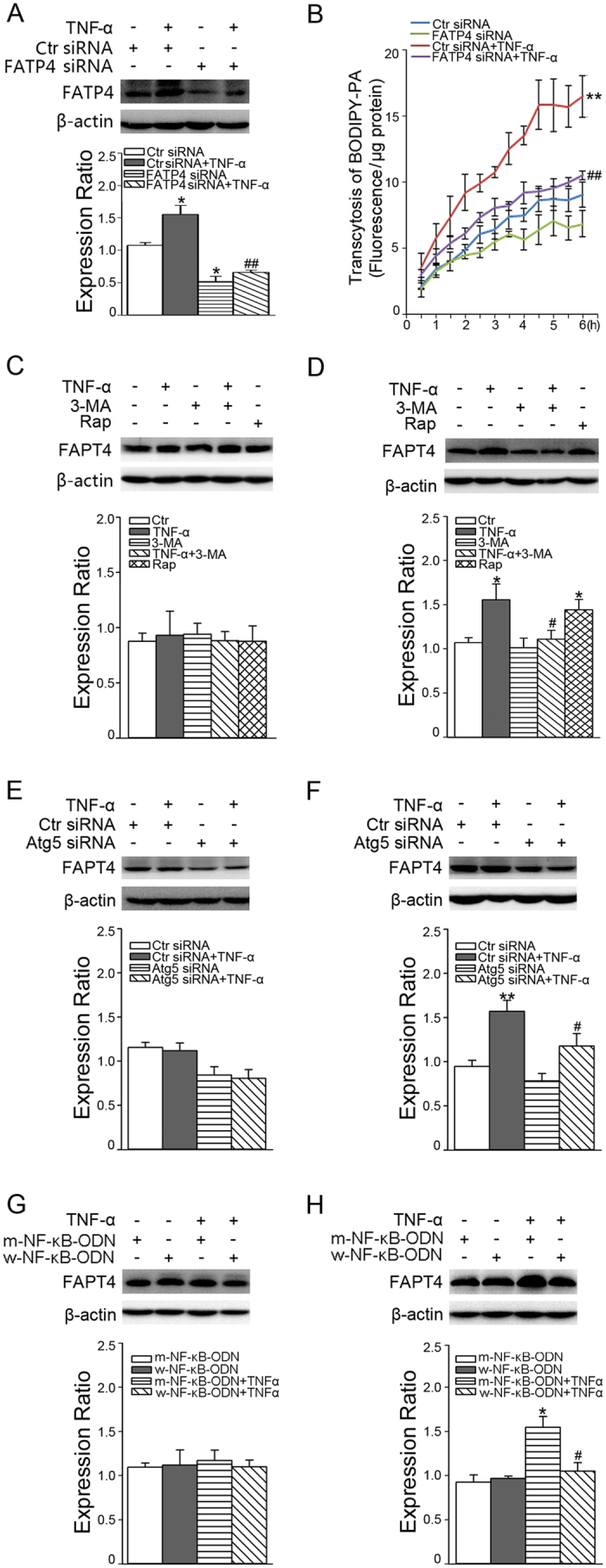
FATP4 mediated TNF-α-stimulated transcytosis and autophagy and NF-κB involvement. CMECs were transfected with FATP4 siRNA for 48 h, followed by 10 ng/ml TNF-α incubation for 18 h. The expression of FATP4 (**A**) and BODIPY-PA transcytosis (**B**) were assayed; *P < 0.05 or **P < 0.01 versus control (Ctr) siRNA; ^#^*P* < 0.05 or ^##^*P* < 0.01 versus Ctr siRNA+TNF-α (n = 3). After pretreatment with 5 mM 3-MA for 30 min, CMECs were exposed to 10 ng/ml TNF-α or 10 nM Rap for 30 min (**C**) or 18 h (**D**), then the expression of FATP4 was measured; *P < 0.05 versus Ctr; ^#^*P* < 0.05 versus TNF-α (*n* = 4). CMECs transfected with Ctr siRNA or Atg5 siRNA for 48 h were exposed to TNF-α for 30 min (**E**) or 18 h (**F**). The expression of FATP4 was measured; **P < 0.01 versus Ctr siRNA; #*P* < 0.05 versus Ctr siRNA+TNF-α (*n* = 4). CMECs transfected with w-NF-κB-ODN or m-NF-κB-ODN for 24 h were exposed to TNF-α for 30 min (**G**) or 18 h (**H**). The expression of FATP4 was measured; *P < 0.05 versus m-NF-κB-ODN; ^#^*P* < 0.05 versus m-NF-κB-ODN+TNF-α (*n* = 4). Protein expression was evaluated by one-way ANOVA and BODIPY-PA transcytosis was evaluated by MNOVA.

CMECs were transfected with FATP4 siRNA to specifically knock down FATP4 expression, which blunted the effect of TNF-α on PA transcytosis (Fig. 4A,B). We then examined the role of autophagy in TNF-α–induced FATP4 expression. As shown, 3-MA pretreatment (Fig. 4D) or Atg5 siRNA (Fig. 4F) inhibited FATP4 expression induced by TNF-α incubation for 18 h. Likewise, Rap treatment for 18 h also upregulated FATP4 expression (Fig. 4D). W-NF-κB-ODN also abolished the increase of FATP4 expression induced by TNF-α treatment for 18 h (Fig. 4H). However, 30 min TNF-α treatment had no obvious effect on FATP4 expression (Fig. 4C,E,G).

### TNF-α activates autophagy in CMECs

**Figure 5:**
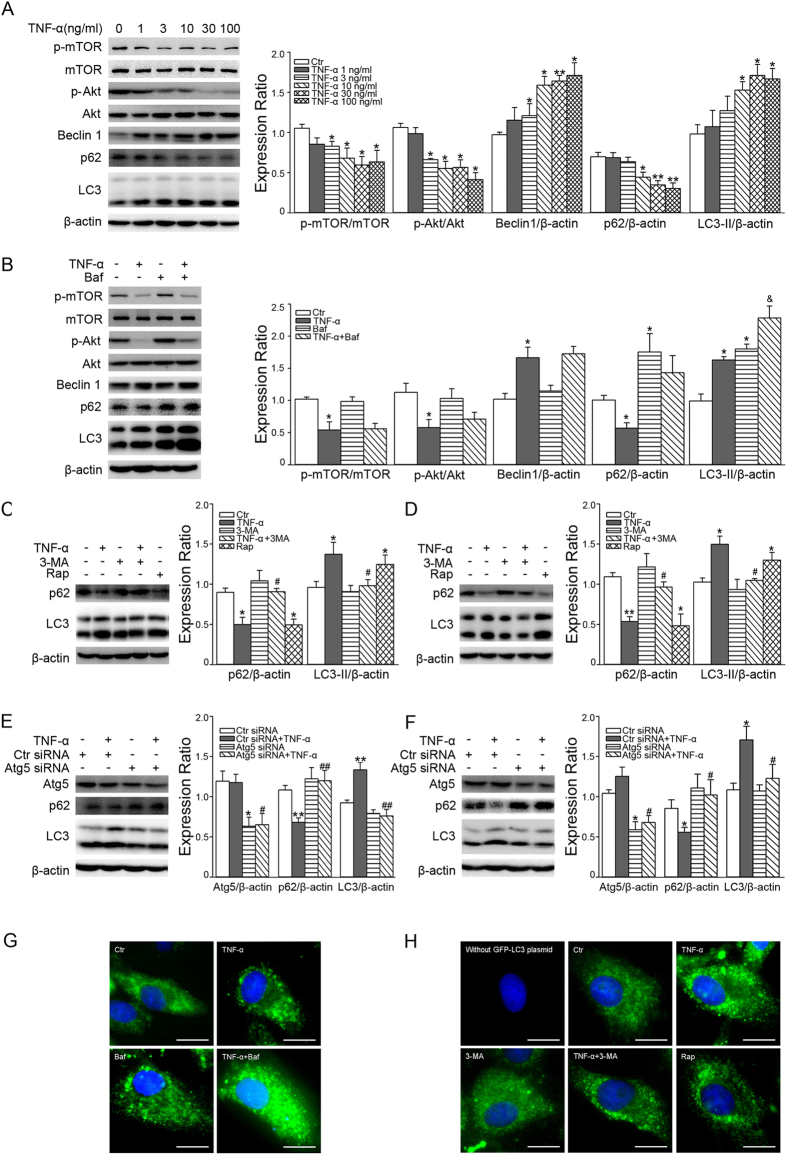
Effect of TNF-α on autophagy in CMECs. (**A**) CMECs were exposed to TNF-α at different concentrations (1, 3, 10, 30 and 100 ng/mL) for 18 h (*n* = 3). (**B**) CMECs were pretreated with 2 nM Baf for 30 min, followed by TNF-α (10 ng/mL) stimulation for 18 h (*n* = 3). After pretreatment with 5 mM 3-MA for 30 min, CMECs were exposed to 10 ng/ml TNF-α or 10 nM Rap for 30 min (**C**) or 18 h (**D**) (*n* = 4). *P < 0.05 or **P < 0.01 versus control (Ctr), ^#^*P* < 0.05 versus TNF-α, ^&^*P* < 0.05 versus Baf. CMECs transfected with Ctr siRNA or Atg5 siRNA for 48 h were exposed to TNF-α for 30 min (**E**) or 18 h (**F**), *P < 0.05 or **P < 0.01 versus Ctr siRNA; ^#^*P* < 0.05 or ^##^*P* < 0.01 versus Ctr siRNA+TNF-α (*n* = 3). Protein expression was evaluated by one-way ANOVA. (**G**) CMECs transfected with GFP-LC3 plasmid were pre-incubated with 2 nM Baf for 30 min, and then exposed to 10 ng/ml TNF-α for 18 h. (**H**) CMECs transfected with GFP-LC3 plasmid were pretreated with 5 mM 3-MA for 30 min, and then exposed to 10 ng/ml TNF-α or 10 nM Rap for 18 h. Scale bars = 10 μm.

We tested the effect of TNF-α on the level of autophagy in CMECs. As shown in Fig. 5A, TNF-α at a concentration of 3 ng/ml decreased the phosphorylation of mTOR and Akt, but increased the expression of Beclin1. In addition, TNF-α 10 ng/ml decreased p62 expression and increased LC3-II expression in the meantime (Fig. 5A). To ensure the activation of autophagy flux induced by TNF-α, we assayed autophagy-associated proteins by western blotting and GFP-LC3 transcription in the presence or absence of Baf, a lysosomal protease inhibitor. Compared with TNF-α alone, the amount of LC3-II expression (Fig. 5B) and the number of GFP-LC3 puncta (Fig. 5G) was further increased in the presence of Baf. Moreover, 3-MA inhibited p62 decrease and LC3-II increase induced by TNF-α treatment for 30 min (Fig. 5C) or 18 h (Fig. 5D), and inhibited LC3 puncta formation induced by TNF-α treatment for 18 h (Fig. 5H). Also, Atg5 siRNA inhibited the effect of TNF-α on p62 and LC3-II (Fig. 5E,F). Furthermore, Rap, an autophagy activator, increased LC3-II expression, as well as LC3 puncta (Fig. 5C,D,H).

### Interaction between autophagy and NF-κB in response to TNF-α stimulation

**Figure 6:**
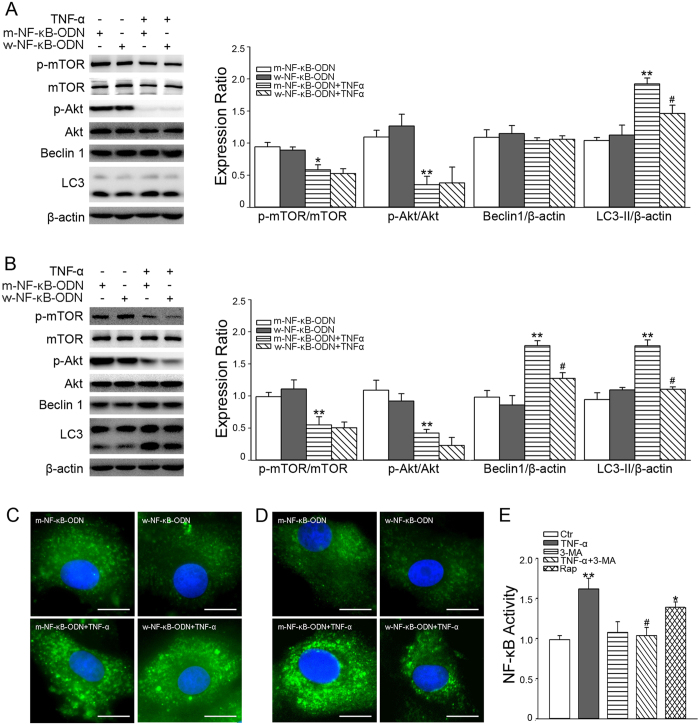
TNF-α stimulates the crosstalk between NF-κB and autophagy. CMECs were transfected with w-NF-κB-ODN or m-NF-κB-ODN for 24 h. Autophagy-associated proteins were analyzed with or without 10 ng/ml TNF-α treatment for 30 min (**A**) or 18 h (**B**). *P < 0.05 or **P < 0.01 versus m-NF-κB-ODN; ^#^*P* < 0.05 versus m-NF-κB-ODN+TNF-α, by one-way ANOVA (*n* = 4). After transfection with ODNs for 24 h, CMECs were transfected with LC3-II plasmid for 24 h. Images of LC3-II puncta are shown after 10 ng/ml TNF-α incubation for 30 min (**C**) or 18 h (**D**). Scale bars = 10 μm. (**E**) CMECs were pretreated with 5 mM 3-MA for 30 min, and then exposed to 10 ng/ml TNF-α; or CMECs were incubated with 10 nM Rap alone for 30 min. NF-κB activity was measured. *P < 0.05 or **P < 0.01 versus control (Ctr); ^#^*P* < 0.05 versus TNF-α, by one-way ANOVA (*n* = 4).

To test the role of NF-κB in TNF-α–stimulated autophagy, CMECs were transfected with w-NF-κB-ODN to specifically antagonize the action of NF-κB. As shown in Fig. 6A and C, w-NF-κB-ODN suppressed the increased expression of LC3-II and formation of LC3-II puncta induced by TNF-α treatment for 30 min. Similarly, as shown in Fig. 6B and D, w-NF-κB-ODN also inhibited the up-regulation of LC3-II expression and LC3-II puncta stimulated by TNF-α treatment for 18 h; in addition, TNF-α-induced Beclin1 upregulation was also attenuated by w-NF-κB-ODN.

To test the role of autophagy in TNF-α–stimulated NF-κB activation, CMECs were pre-treated with 3-MA, followed by exposure to TNF-α. Figure 6E shows that 3-MA inhibited NF-κB activation induced by TNF-α, whereas Rap alone up-regulated NF-κB activity.

### PKCs are involved in TNF-α induced up-regulation of autophagy and NF-κB signaling

**Figure 7:**
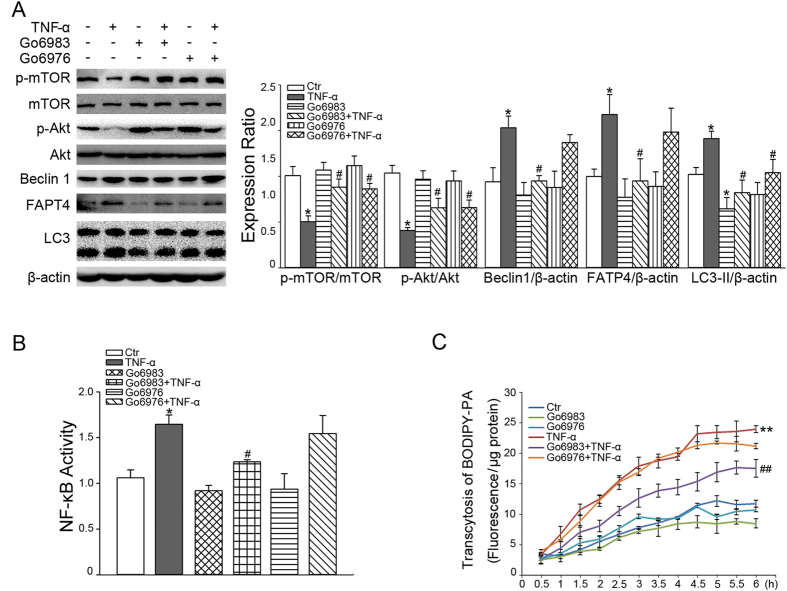
Effects of PKC inhibitors on autophagy, NF-κB activation and PA transcytosis after TNF-α stimulation. CMECs were pre-treated with 15 μM Gö 6983 or 15 μM Gö 6976 for 30 min. (**A**) The cells were exposed to TNF-α for 18 h and the expressions of autophagy-associated proteins and FATP4 were analyzed (*n* = 4). (**B**) The cells were exposed to TNF-α for 30 min and the NF-κB activity was measured (*n* = 3). (**C**) The cells seeded on transwell inserts were exposed to TNF-α for 18 h and BODIPY-PA transcytosis was measured (*n* = 4). *P < 0.05 or **P < 0.01 versus control (Ctr); ^#^*P* < 0.05 or ^##^*P* < 0.01 versus TNF-α. NF-κB activity was evaluated by one-way ANOVA and BODIPY-PA transcytosis was evaluated by MNOVA.

To elucidate the mechanism of TNF-α-incduced autophagy and NF-κB activation, we stimulated cells with a pan-PKC inhibitor, Gö 6983, and a classical PKC inhibitor, Gö 6976. Figure 7A shows that both Gö 6983 and Gö 6976 blunted the decrease of mTOR and Akt phosphorylation and increase of LC3-II stimulated by TNF-α, but only Gö 6983 suppressed the up-regulation of FATP4 and Beclin1 induced by TNF-α. Correspondingly, only Gö 6983 inhibited the NF-κB activation induced by TNF-α (Fig. 7B). Furthermore, only Gö 6983 inhibited the TNF-α-upregulated increase of PA transcytosis (Fig. 7C).

**Figure 8:**
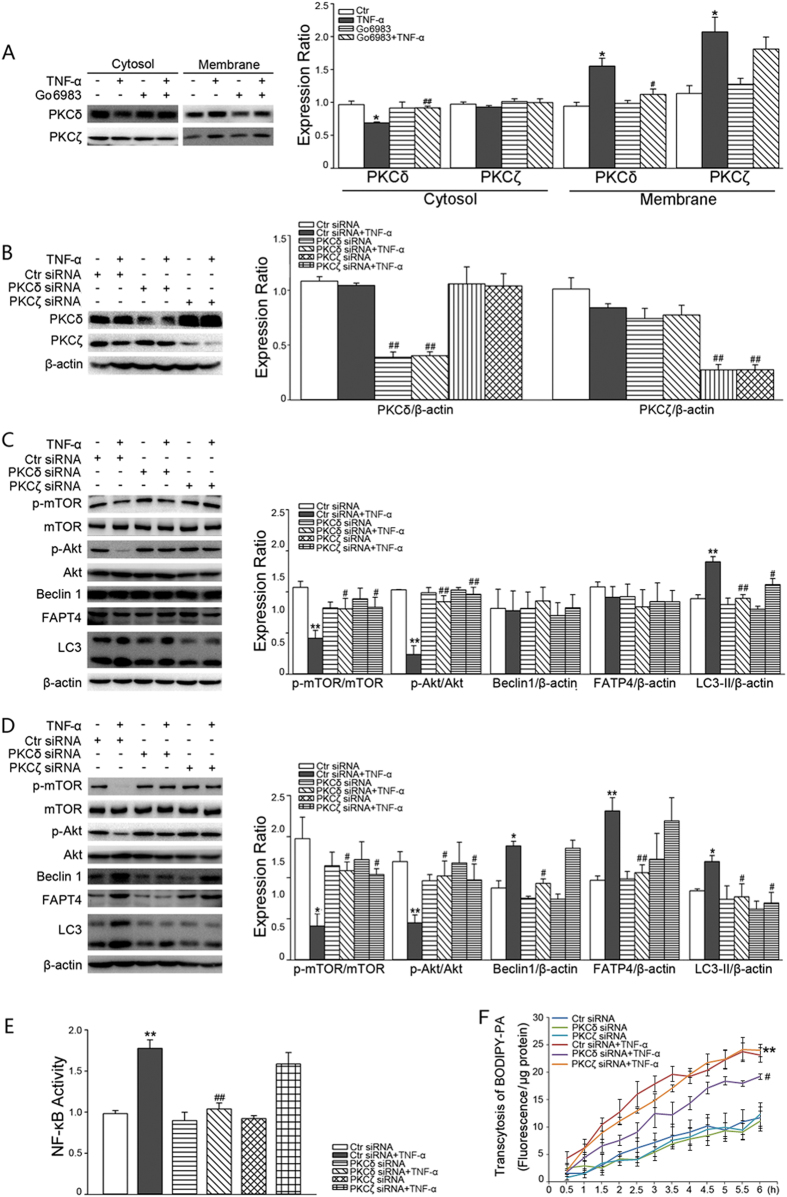
Effects of PKC siRNA on autophagy, NF-κB activation and PA transcytosis after TNF-α stimulation. (**A**) After CMECs were incubated with TNF-α for 30 min with Gö 6983 or Gö 6976 pre-treatment, membrane and cytosol fractions were collected for immunoblotting; *P < 0.05 versus control (Ctr); ^#^*P* < 0.05 or ^##^*P* < 0.01 versus TNF-α (*n* = 3). (**B–F**) CMECs were transfected with PKCδ siRNA or PKCζ siRNA for 48 h. Protein expression was analyzed by immunoblotting after TNF-α treatment for 30 min (**C**) or 18 h (**B,D**) (*n* = 3). NF-κB activity was measured with TNF-α treatment for 30 min (**E**) (*n* = 4). BODIPY-PA transcytosis was measured after TNF-α treatment for 18 h (**F**) (*n* = 4). *P < 0.05 or **P < 0.01 versus Ctr siRNA; ^#^*P* < 0.05 or ^##^*P* < 0.01 versus Ctr siRNA+TNF-α. Protein expression and NF-κB activity were evaluated by one-way ANOVA and BODIPY-PA transcytosis was evaluated by MNOVA.

To explore the specific subtype of PKC involved in these processes, PKCδ and PKCζ siRNA were used. As shown in Fig. 8A, PKCδ and PKCζ translocated into the membrane fraction after TNF-α treatment, and Gö 6983 pre-incubation inhibited this translocation. As shown in Fig. 8B, PKCδ siRNA or PKCζ siRNA specifically knocked down the expression of PKCδ or PKCζ. Experiments with siRNA showed that both PKCδ and PKCζ were involved in the reduced phosphorylation of mTOR and Akt, and activation of autophagy stimulated by TNF-α incubation (Fig. 8C,D). However, only PKCδ defects, but not PKCζ defects, disturbed TNF-α -induced NF-κB activation and PA transcytosis (Fig. 8E,F).

### TNF-α aggravates PA-induced insulin resistance in cardiomyocytes

**Figure 9:**
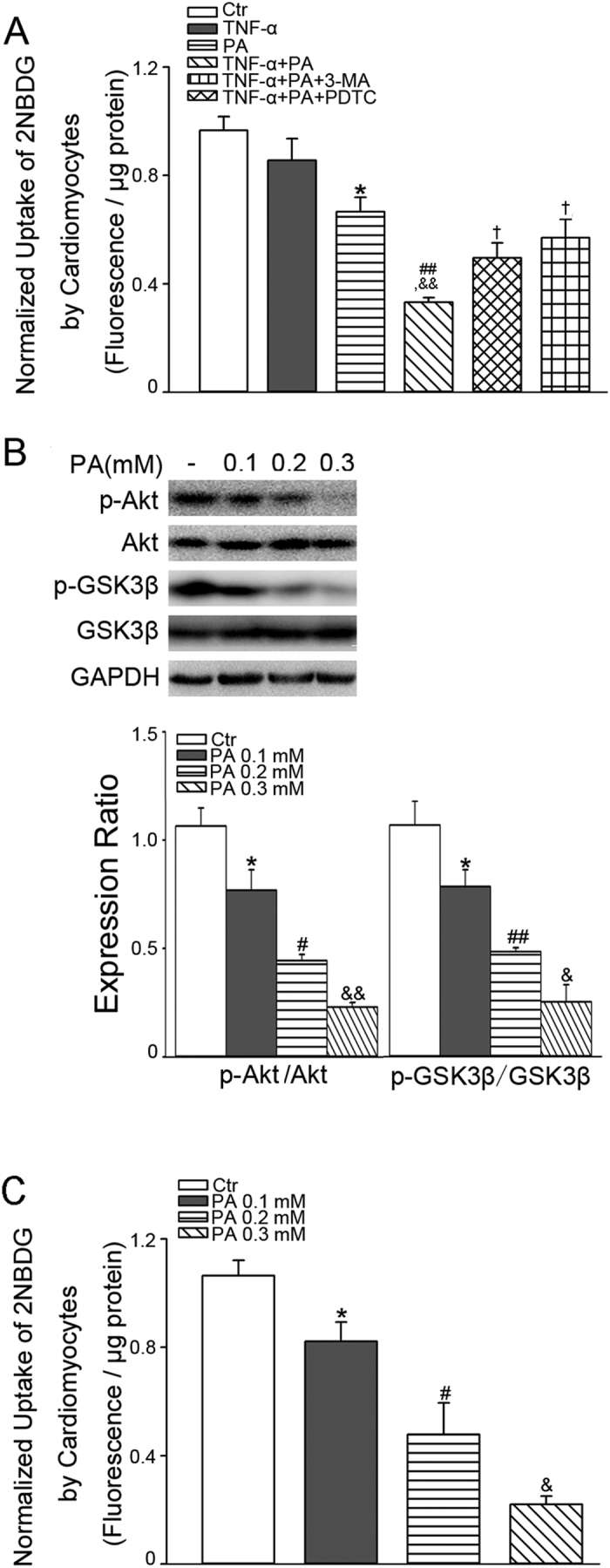
Effect of TNF-α and PA on insulin sensitivity in cardiomyocytes. (**A**) CMECs were seeded in the upper chamber of a transwell and treated with 10 ng/ml TNF-α for 12 h. The medium in the basal chamber was discarded before cardiac myocytes in 300 μl medium were seeded in the lower chamber. PA (400 μM) or BSA was added into the upper chamber for another 24 h. Then cardiomyocytes were incubated with 100 nM insulin in glucose-free DMEM for 10 min and, 2NBDG uptake was measured; *P < 0.05 versus control (Ctr); ^##^*P* < 0.01 versus TNF-α; ^&&^*P* < 0.01 versus PA; ^†^*P* < 0.05 versus TNF-α+PA, by one-way ANOVA (*n* = 4). Following incubation with 0.1, 0.2, or 0.3 mM PA for 23 h, cardiomyocytes were stimulated with 100 nM insulin in glucose-free DMEM for 1 h. (**B**) The phosphorylation of Akt and GSK3β were determined by western blotting. (**C**) 2NBDG uptake was measured after PA incubation with different concentration. *P < 0.05 versus Ctr; ^#^*P* < 0.05 or ^##^*P* < 0.01versus PA 0.1 mM; versus PA 0.1 mM; ^&^*P* < 0.05 or ^&&^*P* < 0.01 versus PA 0.2 mM, by one-way ANOVA (*n* = 4).

To clarify the effect of TNF-α–induced upregulation of PA transcytosis across CMECs on the insulin resistance of myocardia, we used a co-culture model of CMECs and cardiomyocytes to evaluate insulin–induced uptake of 2-[N-(7-nitrobenz-2-oxa-1,3-diaxol-4-yl) amino]-2-deoxyglucose (2NBDG), a fluorescent glucose analogue. The 2NBDG uptake experiment showed that PA transported across CMECs impaired the glucose uptake ability of cardiomyocytes in the presence of insulin, and this effect was potentiated by TNF-α pre-treatment. 3-MA and PDTC blunted the exacerbation effect of TNF-α (Fig. 9A). According to the transport values of BODIPY-PA in the PA transcytosis model, we estimated that PA concentrations in the lower chamber were in the range of 100–300 μM (Fig. 2D). Based on this calculation, we examined cardiomyocyte insulin sensitivity in the presence of various concentrations of PA incubation. As shown in Fig. 9B and C, PA reduced insulin-stimulated Akt and GSK3β phosphorylation as well as 2NBDG uptake in a concentration-dependent manner.

### TNF-α exacerbates PA-induced insulin resistance in mouse heart

**Figure 10:**
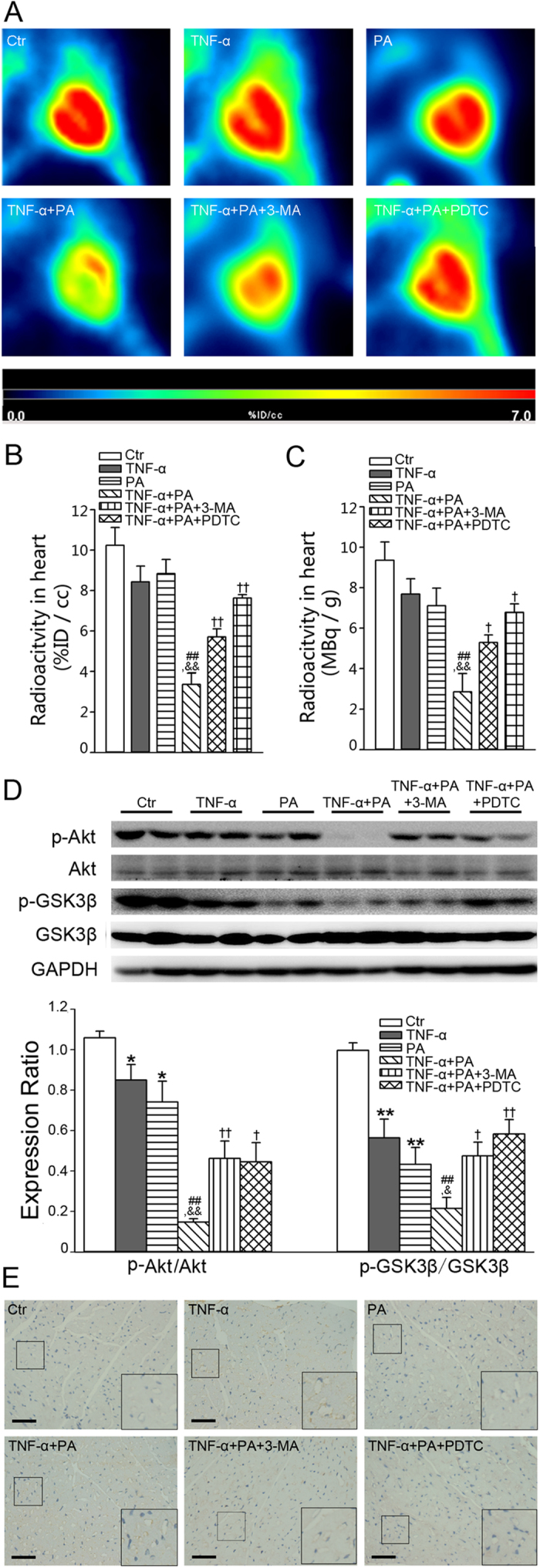
Effect of TNF-α and PA on insulin sensitivity and FATP4 expression in mouse heart. Mice were assigned to five groups: Ctr, TNF-α, PA, TNF-α+PA, TNF-α+PA+3-MA, and TNF-α+PA+PDTC. (**A–C**) PET scanning of mice after PA gavage. Representative PET ^18^F-FDG images (**A**), post-PET measurement of radioactivity in hearts (**B**) and *in vitro* gamma-count heart biodistribution (**C**) are shown. (**D**) Western blot analysis of insulin signaling in hearts. *P < 0.05 or **P < 0.01 versus control (Ctr); ^#^*P* < 0.05 or ^##^*P* < 0.01 versus TNF-α; ^&^*P* < 0.05 or ^&&^*P* < 0.01 versus PA; ^†^*P* < 0.05 or ^††^*P* < 0.01 versus TNF-α+PA, by one-way ANOVA, (*n* = 4). (**E**) Immunohistochemistry for FATP4 expression in hearts.

To verify TNF-α-stimulated PA transcytosis and heart insulin resistance *in vivo*, we assessed myocardial glucose uptake using a small animal PET with ^18^F-fluorodeoxyglucose (FDG). The representative PET images of different groups are shown in Fig. 10A. Figure 10B summarizes the myocardial uptake of ^18^F-FDG obtained from the PET images. Mice in the TNF-α + PA-treated group showed a significant decrease in insulin-stimulated ^18^F-FDG uptake compared with mice treated with TNF-α or PA alone. Pretreatment with 3-MA or PDTC inhibited the reduction of ^18^F-FDG uptake induced by TNF-α+PA treatment. After PET scan, the hearts were rapidly harvested and radioactivity was measured with a gamma-counter. As shown in Fig. 10C, the radioactivity changes were in line with those obtained from the PET scan.

Compared with TNF-α or PA alone, TNF-α+PA treatment markedly reduced the insulin-induced phosphorylation of Akt and GSK3β. 3-MA or PDTC injection enhanced the effect of insulin-stimulated phosphorylation of Akt and GSK3β in mouse hearts exposed to TNF-α+PA treatment (Fig. 10D). Compared to controls, FATP4 expression in heart microvessels was upregulated in the TNF-α group and TNF-α+PA group, while 3-MA or PDTC injection decreased the expression of FATP4 compared to the TNF-α+PA group (Fig. 10E).

## Discussion

During pre-diabetes, myocardia are exposed to a variety of risk factors such as elevated levels of FFA and inflammatory factors, TNF-α, IL-1β, and CRP. Many studies have reported that TNF-α or FFA induce cardiac insulin resistance, and further deteriorate cardiac function via multiple molecular mechanisms. Here we demonstrated that TNF-α significantly up-regulated PA transcytosis across CMECs and promoted myocardial PA uptake. Consequently it exacerbated myocardial insulin resistance. During this process, NF-κB activation and enhanced autophagy flux crosstalk with each other to orchestrate the entire event.

To quantify PA transcytosis across CMECs, we established a modified *in vitro* model. This model is easy to perform, highly sensitive and avoids the use of radioactive components. BODIPY-PA was used as an easily recognizable substitute for PA. The length of BODIPY-PA closely approximates the length of C 16:0^36^. BODIPY-PA mimic FA transport processes and can be competitively inhibited by unlabeled PA^37^. Thus, it was used as an appropriate fluorescent LCFA analogue in the FA uptake assay^23^. In the presence of the continuous capillary endothelium in myocardia, FA cannot pass through the endothelium barrier *in vivo*, but can pass through a cultured CMEC monolayer via the paracellular space. Many studies have reported that the cellular transport of FA is an active protein-mediated process, which is saturable. Therefore, to subtract the amount of paracellular transport, we set a pair of inserts for each group and added 10-fold excess molar PA in the competitive inserts. Transendothelial transport of PA was measured by subtracting the fluorescent intensity of the competitive insert from that of the non-competitive insert. In plasma, FA is bound to proteins such as albumin, and dissociates from the complexes at the surface of capillaries^19^,^38^. The concentration of albumin influences the affinity for PA in the PA transport system. We chose a molar tatio of PA:BSA of 1:0.375 because this molar ratio is consistent with the ratio of combination of PA and BSA^9^,^36^,^39^. By using this model, we found that PA transcytosis across CMECs was concentration- and time-dependent. We also demonstrated that TNF-α significantly increased the transcytosis of PA across CMECs via up-regulating the expression of FATP4.

Our study confirmed that TNF-α activated NF-κB and accelerated autophagy flux, which is consistent with previous studies^40^,^41^. It has been suggested that TNF-α induced autophagy is involved in multiple complex mechanisms. Upregulation of the transcript levels of Atg5, Atg7 and Beclin1 in adipocytes was observed after TNF-α stimulation, and the relationship between the high level of Atg7, Beclin1, and TNF-α was reported in adipose tissue from obese participants^40^. Beclin1 expression was increased in TNF-α-treated cells and was insensitive to Akt inhibitors. However, increased LC3-II expression in TNF-α treated cells was sensitive to Akt inhibitors although TNF-α inhibited Akt phosphorylation^42^. Other studies suggested that TNF-α induced p62-mediated autophagic activity that could be inhibited by 3-MA^43^. Similar results in our study showed that reduced phosphorylation of mTOR and increased expression of Beclin1 participated in TNF-α-induced autophagy, reflected by decreased levels of p62 expression, increased levels of LC3-II expression and increased numbers of LC3 puncta. As described previously, we also compared the amount of LC3-II among samples because the sensitivity of LC3-II by anti-LC3 antibody is much higher than that of LC3-I^44^.

To further determine the role of NF-κB and autophagy in TNF-α–stimulated PA transcytosis, w-NF-κB-ODN, a specific NF-κB inhibitor, and two autophagy inhibitors with a different structure and mechanism were used. It was found that TNF-α-stimulated PA transcytosis was largely inhibited by NF-κB inhibitor and autophagy inhibitors. Activation of autophagy by Rap also substantially enhanced PA transcytosis across CMECs. These results suggested the critical roles of these two processes in mediating the TNF-α–stimulated PA transcytosis.

PA-induced insulin resistance is associated with mitochondrial dysfunction, ROS production and phosphorylation of the insulin receptor substrate-1^45^,^46^. In the present study, we also evaluated the synergistic effect of TNF-α and PA on cardiac insulin sensitivity *in vitro* and *in vivo*. In an *in vitro* CMEC and cardiomyocyte co-culture model, we confirmed TNF-α promoted PA-induced insulin resistance and this effect was positively correlated with the amount of PA. In mice, TNF-α and PA were administered to mimic risk factors during pre-diabetes and insulin-stimulated ^18^F-FDG uptake in the heart was evaluated by PET. We found that TNF-α and PA synergistically reduced insulin- stimulated ^18^F-FDG uptake in the heart and increased the expression of FATP4 in myocardial capillaries. Changes in ^18^F-FDG uptake and FATP4 expression induced by TNF-α and PA were improved not only by autophagy inhibitor 3-MA but also by NF-κB inhibitor PDTC. These observations further support the *in vivo* significance of the inhibition of NF-κB or autophagy, as strategies for the prevention of insulin resistant or treatment of pre-diabetes.

To illustrate how NF-κB and autophagy were involved in TNF-α-stimulated PA transcytosis, we studied the role of NF-κB and autophagy in TNF-α-induced FATP4 expression. The FATP gene contains binding sites for transcriptional factor NF-κB^21^. In this study both w-NF-κB-ODN and autophagy inhibition (3-MA and Atg5 siRNA) reduced the elevated FATP4 expression induced by TNF-α, whereas activation of autophagy by Rap alone up-regulated FATP4 expression. These results suggest that both NF-κB and autophagy signaling pathways participate in TNF-α-stimulated FATP4 expression. We also found that inhibition of NF-κB by w-NF-κB-ODN inhibited TNF-α–stimulated autophagy. Moreover, inhibition of autophagy by 3-MA and Atg5 siRNA alleviated TNF-α–stimulated NF-κB activation, which suggested that NF-κB activation and enhanced autophagy flux may crosstalk with each other during TNF-α–stimulated FATP4 expression. According to reports in recent years, autophagy is intricately interconnected with the NF-κB system via various mechanisms. Mergny *et al*. found that NF-κB activation mediated repression of autophagy in TNF-α-treated Ewing sarcoma cells^47^. Chang *et al*. reported that autophagy directly controlled NF-κB via degradation of NF-κB RELA/p65^48^. Distinct autophagic triggers, including starvation, and Rap lead to the activation of IKK, followed by the phosphorylation-dependent degradation of Iκ-Bα and nuclear translocation of NF-κB^49^. In addition, Atg5-, Atg7- and Beclin1 depletion abolished TNF-α-induced NF-κB activation^41^. Ghosh *et al*. considered that mTOR inhibited NF-κB activation induced by TNF-α in TSC-1/2 deficient MEFs, which was restored by Rap-mediated inhibition of deregulated mTOR activity^50^. Autophagy receptor p62 also plays an essential role in the canonical NF-κB pathway^41^. In addition, NF-κB family member, p65 upregulated Beclin1 mRNA and protein levels, coupled to increased autophagy^49^,^51^. These findings are consistent with our observation that there is an intimate crosstalk between NF-κB signaling and autophagy.

We further attempted to determine the molecular pathway involved in TNF-α stimulated NF-κB and autophagy. PKC is a family of phospholipid-dependent serine/threonine kinases centrally involved in the spatial control of signal transduction in cells. Specified by their divergent regulatory domains, the PKC family can be divided into three structurally and functionally distinct subclasses: classical PKC isozymes (cPKC), novel PKC (nPKC) and atypical PKC (aPKC). PKC functions as a negative regulator in Akt signaling pathway^52^ and as a positive regulator in NF-κB activation^53^. PKC positively regulated oridonin-induced Beclin1 expression and autophagy^54^. According to our data, PKC is an intracellular signaling mediator of the actions of TNF-α^55^. We conducted a preliminary screening using two inhibitors, Gö 6983, a pan-PKC inhibitor, and Gö 6976, a cPKC inhibitor. We found that Gö 6983 significantly inhibited the TNF-α-induced decrease of Akt and mTOR phosphorylation, the increase of Beclin1, LC3-II and FATP4 expression, the activation of NF-κB, as well as the up-regulation of PA transcytosis. In contrast, Gö 6976 had no effect on Beclin1 and FATP4 expression, NF-κB activity and PA transcytosis, but only a slight effect on LC3-II levels, although Gö 6976 inhibited the decrease of Akt and mTOR phosphorylation. Considering the insignificant effect of cPKC, we then focused on the other subtypes, novel PKC (nPKC) and atypical PKC (aPKC), which might play an important role in TNF-α-induced up-regulation of NF-κB activity and autophagy flux, as well as PA transcytosis. Novel PKC subtypes were shown to be involved in autophagy^55^. PKCδ is required for TNF-α initiated NF-κB activation^56^. An atypical PKC, PKCζ, an aPKC has emerged as a pathologic mediator of endothelial cell dysfunction, and is important in control of TNF-α induced transcriptional activity of NF-κB and the expression of NF-κB-dependent genes^57^. Our results indicated an increase mainly in the particulate fraction of PKCζ, and the obvious translocation of PKCδ from the cytosolic to particulate fraction after TNF-α treatment. This is consistent with previous observations^55^,^58^. We then explored the role of individual PKC isoforms in TNF-α-induced transcytosis utilizing PKCδ or PKCζ siRNA. TNF-α treatment results in the translocation of PKCδ and PKCζ to membrane fractions. However, only PKCδ was involved in TNF-α-induced FATP4 expression and PA transcytosis, although both PKCδ and PKCζ participated in autophagy activation. This may account for a very weak level of translocation of PKCζ in response to TNF-α^58^. PKCδ mainly participated in TNF-α-induced PA transcytosis via activating autophagy and NF-κB, while PKCζ was involved in TNF-α–stimulated autophagy.

**Figure 11:**
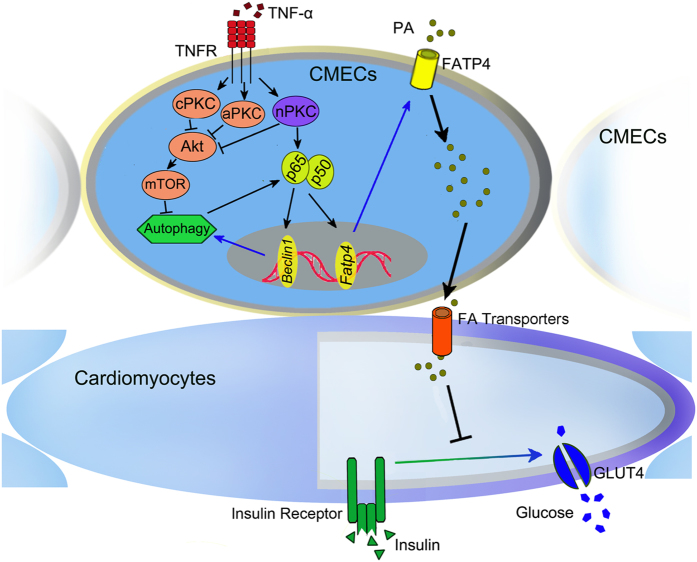
Schematic diagram of TNF-α -induced PA transcytosis in CMECs and the effect of PA on insulin sensitivity in cardiomyocytes.

As summarized in Fig. 11, the present study elucidated the remarkable role of TNF-α in increasing PA transport across the endothelium barrier, which in turn facilitates PA-related cardiac insulin resistance. The mechanism of TNF-α-stimulated PA transcytosis involves PKC activation, crosstalk between NF-κB and autophagy, and finally FATP4 expression. Our study indicates that TNF-α signaling is implicated in cardiac insulin sensitivity regulation, further emphasizing promising therapeutic targets for disease conditions with chronic low-grade inflammation, such as metabolic cardiovascular syndrome.

## Materials and Methods

### Isolation and culture of rat CMECs and cardiac myocytes

CMECs and cardiac myocytes were isolated as previously described^59^. Adult male Sprague-Dawley rats (200–250 g) were heparinized (heparin sodium, 1U/kg body weight, i.p.) and anesthetized (urethane, 1 g/kg, i.p.). The heart was removed and washed in ice-cold PBS, followed by dipping into 75% ethanol for 10 s to remove potential bacterial contaminations and to devitalize the epicardium mesothelia cells. After atria and valves were discarded, ventricular tissue was minced into 1 mm^3^ pieces and resuspended in 10 ml modified D-Hanks (supplemented with glucose 2 mg/ml, taurine 2.5 mg/ml, and BSA 0.1%) (Taurine, Sigma, USA). Tissues were pre-digested for 5 min in 10 ml of pre-warmed collagenase (1 mg/ml collagenase type II suspended in modified Hank’s buffer) (Invitrogen, Grand Island, NY, USA), sheared by passing 10 times through a 10 ml pipette and supernatant removed. Tissues were digested twice more with10 ml collagenase for 10 min with shearing every 5 min. Supernatants from each digestion were removed into a new tube containing 2 ml FBS (Gibco, Australia) to stop digestion. The tissue pellet at this step was used for CMECs isolation. Supernatants with 2 ml FBS were centrifuged at 25 × g for 3 min, and the cell pellet obtained was resuspended in 10 ml HBSS containing 2 ml FBS. Cardiac myocytes were separated from debris and non-myocytes by gravity sedimentation through a 6% BSA cushion at room temperature for 15–20 min. Myocyte viability was examined by trypan blue exclusion, and their identity was confirmed as a typical rod-shaped, striated appearance.

The tissue pellet obtained after myocyte isolation was then digested using trypsin EDTA buffer (0.05% trypsin, 0.1 mmol/L EDTA, enriched with 2.0 mg/ml glucose and 1.0 μg/ml DNase) for 10 min at 37 °C, and centrifuged at 300 rpm for 3 min. The supernatants were collected. After a total of three 10 min digestion periods, all supernatant was added into DMEM supplied with 10% FBS and centrifuged at 1000 rpm for 7 min. The cells were resuspended in 3 ml DMEM (Hyclone, Logan, UT) containing 10% FBS, 100 U/ml penicillin, 100 U/ml streptomycin, 30 ng/ml ECGS (Sigma, USA), and 10 U/ml heparin. Then cells were seeded into collagen-coated T25 flasks (Corning, USA) and were set to passage 0. The first subculture was set to passage 1. The cells were used within passage 1~4. CMECs were identified with immunofluorescent staining for vWF and DiI-AcLDL uptake (Details were shown in Supplemental data).

### BODIPY-PA transcytosis

Two mg/ml stock of BODIPY-PA (Invitrogen, USA) in 200-proof ethanol was dried with nitrogen gas, and then resuspended with DMEM containing 1% FA free BSA (FF-BSA, Sigma, USA) to a final concentration of 400 μM. PA was dissolved in DMEM containing 10% FF-BSA for 4 mM. The ratio of BODIPY-PA or PA: BSA was 1:0.375, and this ratio is suitable for FA uptake^39^.

As shown in Fig. 1, the *in vitro* model used to measure PA transcytosis was modified from previous methods^23^,^60^,^61^,^62^. A LCFA analogue, BODIPY-PA was used to quantify the cellular transport of PA. Transport of BODIPY-PA trough endothelial cells is similar to PA and can be competitively inhibited by PA^37^. CMECs were seeded on a 24-well cell culture inserted costar transwell (6.5 mm diameter and 0.4 μm pore size) to form an integrated cell monolayer. The integrity of the cell monolayer was tested as described previously^60^,^61^.

Two inserts of cell monolayers with equal integrity were assigned into one paired group, which contained a noncompetitive insert and a competitive insert, respectively. BODIPY-PA (final concentration 40 μM with 0.1% BSA) was added to the apical chamber of the noncompetitive insert, while BODIPY-PA (final concentration 40 μM with 0.1% for BSA) and a tenfold-excess PA (final concentration 400 μM with 1% for BSA) were added to the apical chamber of the competitive insert. The same concentration of FF-BSA as that in the upper chamber of the transwell was added into the lower chamber in both the noncompetitive and competitive inserts. Then 10% FF-BSA was added to the lower compartments to achieve the same concentration with the apical chamber. Finally, 100 μl samples were collected from the lower compartment every 30 min during the BODIPY-PA incubation period, and replaced with 100 μl medium containing the same concentration of FF-BSA. After 6 h, fluorescence of all collected samples was detected via a fluorescence spectrophotometer (Tecan, Ininite F200PRO) with excitation and emission wavelengths of 490 nm and 520 nm, respectively. Background fluorescence determined by DMEM was subtracted from the value of each sample. Transport amounts at each time point were calculated by the following formula: Transport amount of time point n = (Fluorescence value of time point n * 6) − (Fluorescence value of time point (n − 1) *5). Transport values at each time point were equal to the sum of values of the time points before the current time point. Transcytosis of BODIPY-PA was calculated by subtracting paracellular transport (the competitive insert) from total transport (the noncompetitive insert). The final value was normalized by protein concentrations of CMECs on each insert. A standard curve on fluorescence value was constructed by BODIPY-PA concentration: 5, 2.5, 1.25, 0.625, 0.3125, 0.15625, 0.078125, 0.039063, and 0 nmol/100 μl.

### Western Blotting analysis

The protein samples were separated by SDS-PAGE gel and then electrotransferred to PVDF membranes (Millipore, USA). Subsequently, blots were subjected to immunostaining with the following primary antibodies against phospho-GSK3β (Ser9), β-actin, Beclin-1, phospho-mTOR (Ser2448), mTOR, p62 and LC3 (Cell Signaling Technology, Beverly, MA, USA); phospho-Akt (Ser473) (EPITOMICS, Burlingame, CA, USA); Akt (ABclonal Technology, ABclonal Biotech Co., Ltd., USA); and GSK3β, Atg5, FATP4 (Proteintech, China) were used at 1:1000 dilution. Goat anti-rabbit or goat anti-mouse secondary antibodies (Abbkine, Redlands, CA, USA) were used at 1:10,000 dilutions. Protein samples were obtained from cell or tissue lysates. Membrane and cytosol fraction isolation was performed according to kit instructions (Proteintech, China), and then protein concentrations were adjusted to be same among groups.

### NF-κB activity assay

NF-κB activity was assayed by an ELISA-based method as described previously^63^,^64^. Cell lysates were centrifuged at 14,000 rpm for 20 min and the supernatant was collected. After quantification with BCA reagent (Pierce, Rockford, IL), the concentration of proteins in cell extracts were adjusted to 1.5 μg/μl. Then, the cell extracts were incubated in a 96-well plate coated with the oligonucleotide containing the NF-κB consensus-binding site (5′-GGGACTTTCC-3′). Activated transcription factors from extracts specifically bound to the respective immobilized oligonucleotide. The activities were then detected with the primary antibody to NF-κB p65 (1:500, Proteintech, China). The activities were finally determined as absorbance values measured with a microplate reader at a wavelength of 450 nm.

### GFP-LC3 transfection

CMECs were transfected with GFP-LC3 plasmid for 48 h using Effectent transfection reagent (Qiagen, Hilden, Germany) according to the manufacturer’s instruction. GFP-LC3 plasmid was a gift from Ruiguang Zhang (Cancer Center, Union Hospital, Tongji Medical College, Huazhong University of Science and Technology)^65^. Images were obtained with a fluorescence microscope.

### siRNA transfection

CMECs were transfected with 10 nM FATP4 siRNA or scramble siRNA (Ctr siRNA) using Hiperfect transfection reagent (Qiagen, Hilden, Germany) for 48 h according to the manufacturer’s instructions. siRNAs were synthesized by Guangzhou Ribobio, China. The FATP4 siRNA sequence was as follows: 5′-AACAAGAAGATTGCTAGTGAT-3′^66^. The Atg5 siRNA sequence was as follows: 5′-GACGCUGGUAACUGACAAATT-3′^67^. The PKCδ siRNA sequence was as follows: 5**′**-AAAAGGCAAATTCACAAACAG-3**′**^68^. The PKCζ siRNA sequence was as follows: 5**′**-GGUUGUUCCUGGUCAUCGAtt-3**′**^69^.

### Decoy Oligonucleotide (ODN) transfection

Wide-type-NF-κB decoy ODN (w-NF-κB-ODN) and mutant-NF-κB decoy ODN (m-NF-κB-ODN) were synthesized by Invitrogen China (Shanghai, China) and labeled by 7-Amino-4-methylcoumarin (AMC). Their sequences are as follows^70^: w-NF-κB-ODN 5**′**-GAA ATT TCA GGG AAA TCC CTT CAA GAA AAC TTG AAG GGA TTT CCC T-3**′**; m-NF-κB-ODN, 5**′**-GAA ATT TCA GGG AAA TTA CTT CAA GAA AAC TTG AAG TAA TTT CCC T-3**′**. These ODN were annealed for 8 h, while the temperature decreased from 80 °C to 25 °C. Following the addition of T4 ligase (1U), the mixture was incubated for 18 h at 16 °C to generate a covalently ligated ring-type decoy molecule. CMECs were transfected with 200 nM NF-κB or m-NF-κB decoy ODN using Oligofectaminet ™ Reagent (Invitrogen, Carlsbad, CA) for 24 h according to the manufacturer’s instructions.

### Measurement of 2NBDG uptake

Glucose uptake activity was measured in primary cardiac myocytes using a fluorescent D-glucose analogue 2-[N -(7-nitrobenz-2-oxa-1,3-diaz-ol-4-yl)amino]-2-deoxy-D-glucose (2-NBDG) (Cayman Chem, MI, USA). After washed with glucose-free DMEM, cardiac myocytes were incubated with 100 nM insulin in glucose-free DMEM for 10 min, and then 2NBDG with a 50 μM final concentration were added for 50 min. Cells were lysed and fluorescence values of cell lysates were measured via a fluorescence spectrophotometer (Tecan, Ininite F200PRO) with excitation and emission wavelengths of 490 nm and 520 nm, respectively. The fluorescence value was normalized by protein concentration of cells.

### Myocardial PET

Nine-week-old non-starved male C57BL6/J mice underwent PET on a Trans-PET^®^ BioCaliburn^®^700 system^23^. Mice were randomly assigned to six treatment groups: Ctr, TNF-α, PA, TNF-α+PA, TNF-α+PA+3-Methyladenine (3-MA, Sigma, USA) or TNF-α+PA+ Pyrrolidinedithiocarbamic acid (PDTC, Sigma, USA). Mice in PA, TNF-α+PA, TNF-α+PA+3-MA or TNF-α+PA+PDTC groups were given PA (0.015 g PA/g body weight per day) dissolved in 0.5% PEG for 3 days. Others were given the same volume of PEG^9^. At the fourth day, PA or PEG was given 1 h before PET scan. TNF-α treated mice were given TNF-α (20 μg/kg, i.p.) 12 h before PET scan. Others were given PBS containing 0.1% BSA as vehicle control. 3-MA or PDTC treated mice were pre-performed with 3-MA (15 mg/kg) or PDTC (200 mg/kg) 1 h before TNF-α. Mice were warmed for 10 min under a heat lamp after injection of insulin (1 U/kg, i.p., Roche) and ^18^F-FDG, and then anaesthetized with isoflurane (5% initially and 1.5% to maintain anesthesia). A whole-body PET acquisition was performed to obtain heart ^18^F-FDG uptake values and calculate the percentage of the injected dose per cc (%ID/cc). After the PET scan, animals were euthanized. Hearts were harvested and washed in ice-cold PBS and excess liquid was removed. The radioactivity was measured using a well gamma-counter (Wizard2 Gamma Counter, PerkinElmer) and hearts were weighed. Cardiac ^18^F-FDG uptake was then expressed as the %ID/cc obtained by calculating the ratio of myocardial ^18^F-FDG activity to injected dose normalized to the body and heart weights.

### Histological analysis

For immunohistochemical analysis, hearts were rapidly fixed in 4% paraformaldehyde solution. After embedded in paraffin, they were cut into 5-μm-thick sections and stained with FATP4 antibody (Proteintech, China) at a 1:50 concentration followed by counter staining with hematoxylin.

### Statistical Analysis

All data are expressed as the mean ± SEM from at least three separate experiments. Individual group statistical comparisons were analyzed by unpaired Student t-test; and multiple-group comparisons were evaluated by one-way ANOVA with post-hoc testing; multiple-group comparisons in transcytosis experiments were evaluated by MNOVA. A value of *P* < 0.05 was considered statistically significant.

### Study approval

All the experimental procedures were performed in accordance with the International Guidelines for Care and Use of Laboratory Animals and approved by the Animal Ethical Committee of Tongji Medical College, Huazhong University of Science and Technology.

## Additional Information

**How to cite this article**: Li, W. *et al*. TNF-α stimulates endothelial palmitic acid transcytosis and promotes insulin resistance. *Sci. Rep.* **7**, 44659; doi: 10.1038/srep44659 (2017).

**Publisher's note:** Springer Nature remains neutral with regard to jurisdictional claims in published maps and institutional affiliations.

## Supplementary Material

Supplementary Material
