# Supplementary material for: TNF-α stimulates endothelial palmitic acid transcytosis and promotes insulin resistance
Source: Sci Rep. 2017 Mar 17;7:44659. doi: 10.1038/srep44659 (PMC5356338; doi:10.1038/srep44659)
Supplement: Supplementary Material [file srep44659-s1.doc]

TNF-α stimulates endothelial palmitic acid transcytosis and promotes insulin resistance

Wenjing Li^b,#^, Xiaoyan Yang^b,c,#^, Tao Zheng^b^, Shasha Xing^b^, Yaogong Wu^b^, Fang Bian^b^, Guangjie Wu^b^, Ye Li^b^, Juyi Li^b^, Xiangli Bai^a^, Dan Wu^b^, Xiong Jia^a^, Ling Wang^b^, Lin Zhu^a^, Si Jin^a,b,*^


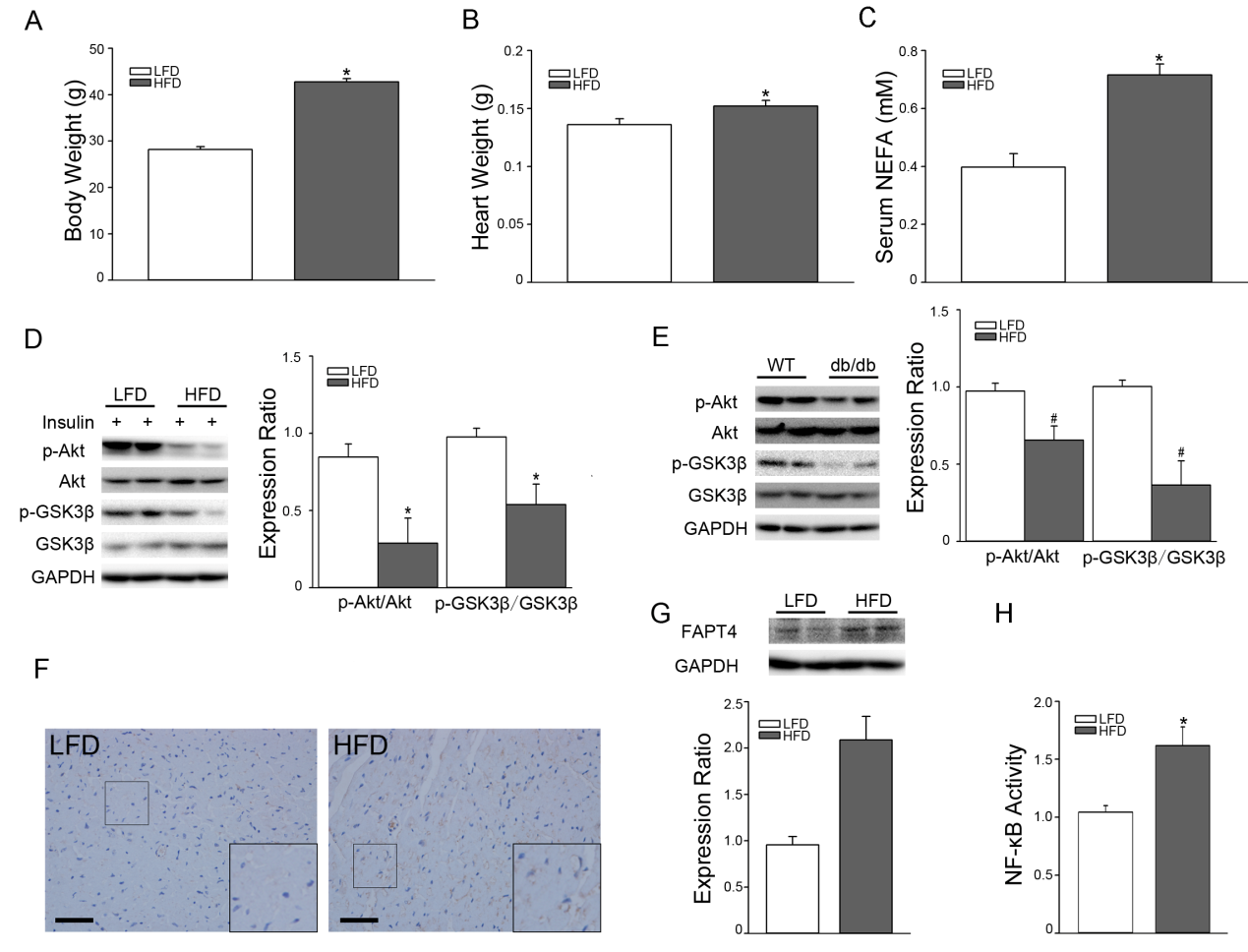


**Figure S1: Indexes in T2D mice.** After high fat diet or low fat diet for 4 months, mouse body weight (A), heart weight (B) and serum non-esterified FA (NEFA) concentrations were measured (C). Western blotting analysis for insulin signaling in HFD-mice (D) and db/db mice (E). Immunohistochemical (F) and western blotting analysis (non-insulin treated mice) for FATP4 expression (G). NF-κB activity assay (H). **P*< 0.05 versus LFD; *n=5*. #*P*< 0.05 versus WT, by 2-tailed Student’s test ( *n*=5).


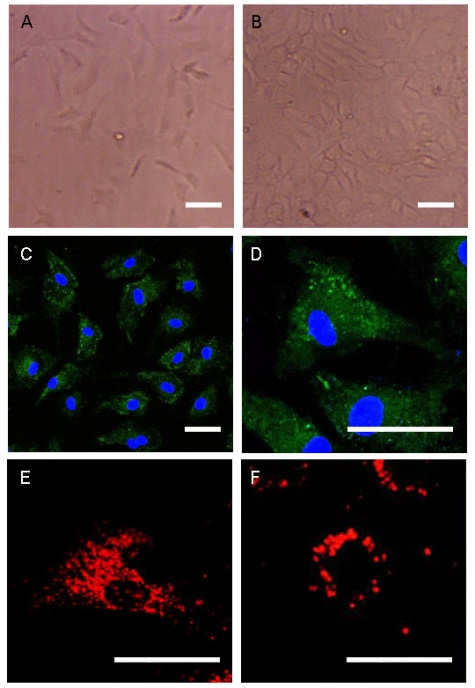


**FigureS2. Morphology and immunofluorescence images of CMECs.** (A, B) Primary CMEC exhibiting a elongated shape (A) when spread as single cells and a irregular “cobblestone” pattern at confluence (B). (C, D) Immunofluorescence images of CMECs showing vWF (green) and cell nuclei stained with DAPI DIPY (blue). (E, F) Dil-Ac-LDL uptake in CMECs (E) or in HUVECs (F).Scale bars =20 μm.
